# Controllable unidirectional transport and light trapping using a one-dimensional lattice with non-Hermitian coupling

**DOI:** 10.1038/s41598-020-58018-2

**Published:** 2020-01-24

**Authors:** Lei Du, Yan Zhang, Jin-Hui Wu

**Affiliations:** 1 Center for Quantum Sciences and School of Physics, Northeast Normal University, Changchun, 130024 China; 2 Beijing Computational Science Research Center, Beijing, 100193 China; 3 Scuola Normale Superiore, 56126 Pisa, Italy

**Keywords:** Optical physics, Quantum optics

## Abstract

We propose a one-dimensional tight-binding lattice with special non-Hermitian coupling, the imaginary part of which is modulated by an effective Peierls phase arising from the synthetic magnetic field. Such a non-Hermitian lattice supports robust unidirectional transport that is reflectionless and immune to defects; it thus can serve as a frequency-selectable light filter. To achieve more applications, we further construct two well-designed structures involving this lattice, namely a heterostructure and a sandwich structure. An optical diode can be realized using the heterostructure, while tunable light trapping and reversal can be realized through phase modulations on the sandwich structure. The results in this paper may not only open up a new path for unconventional light transport but also have potential applications for optical communication.

## Introduction

Controllable light transport has long been an important research objective due to its significant potential in practical applications^1^. In particular, unidirectional transport, which can be used to realize optical isolators and circulators, plays a key role in modern optics^2–13^. Generally speaking, unidirectional light transport can be observed in an asymmetric hybrid system^2^, especially via introducing the nonlinearity^3–7^, where the unidirectionality arises from the synergy between the asymmetry and the nonlinearity. Alternatively, unidirectional light transport can be realized in a two- or three-dimensional photonic system with topological protection^8–12^. As is well known, topologically protected edge (surface) states, which are guided by synthetic gauge fields and propagate along the boundaries of systems, exhibit prominent advantages owing to their robustness, i.e., their immunity to disorders and defects. Such schemes, however, can be implemented only in two- or higher-dimensional photonic systems.

On the other hand, non-Hermitian lattices have attracted considerable research attention in recent years because they facilitate the observation of many novel phenomena that are absent in Hermitian cases, such as non-Hermitian induced delocalization in disorder lattices^14–21^, invisible defects and potentials^22–24^, topological phase transitions^25–28^, anomalous edge states^29,30^, and non-Hermitian induced flat bands^31–34^. More importantly, well-designed non-Hermitian lattices can serve as a powerful platform for realizing unidirectional light transport^35–40^, while common Hermitian ones require various special effects^3,4,7– 11,41,42^. For instance, unidirectional non-Hermitian induced transparency has been realized in a one-dimensional non-Hermitian lattice^35,36^. In that work, imaginary gauge fields, which are achieved by exploiting auxiliary ring resonators with gain and loss media in different half perimeters, are introduced to obtain non-reciprocal hopping rates. Thus, waves are amplified along a propagating direction and undergo attenuation in the opposite direction. Such schemes make robust unidirectional light transport possible and may have applications in directional amplification. However, they suffer from poor tunability. Subsequently, a seminal work^43^ demonstrated that a zigzag lattice with imaginary (non-Hermitian) next-nearest-neighbor coupling supports *tunable* light transport because the coupling is modulated by the effective Peierls phase; however, the transport is bidirectional so that the model lacks non-reciprocity. In this context, combining non-Hermitian induced unidirectionality with tunability may be useful for exploring more novel transport phenomena.

In this paper, we reveal that a class of non-Hermitian one-dimensional lattices can support tunable robust unidirectional transport that is reflectionless and immune to defects. Specifically, the coupling is non-Hermitian with the imaginary part being modulated by a synthetic magnetic field. We consider a well-designed dimerized sawtooth lattice as a potential implementation of the non-Hermitian lattices and show that it is possible to realize by adjusting the synthetic magnetic field a frequency-selectable filter, i.e., one can select the wave number of the outgoing wave. Furthermore, we construct two structures involving the non-Hermitian lattice: a heterostructure and a sandwich structure. The former supports unidirectional transmissionless transport and thereby can be used as an optical diode. The latter can be used to realize controllable light trapping and reversal, where the trapping duration and region can be controlled readily with high efficiency.

## Model and Methods

**Figure 1:**
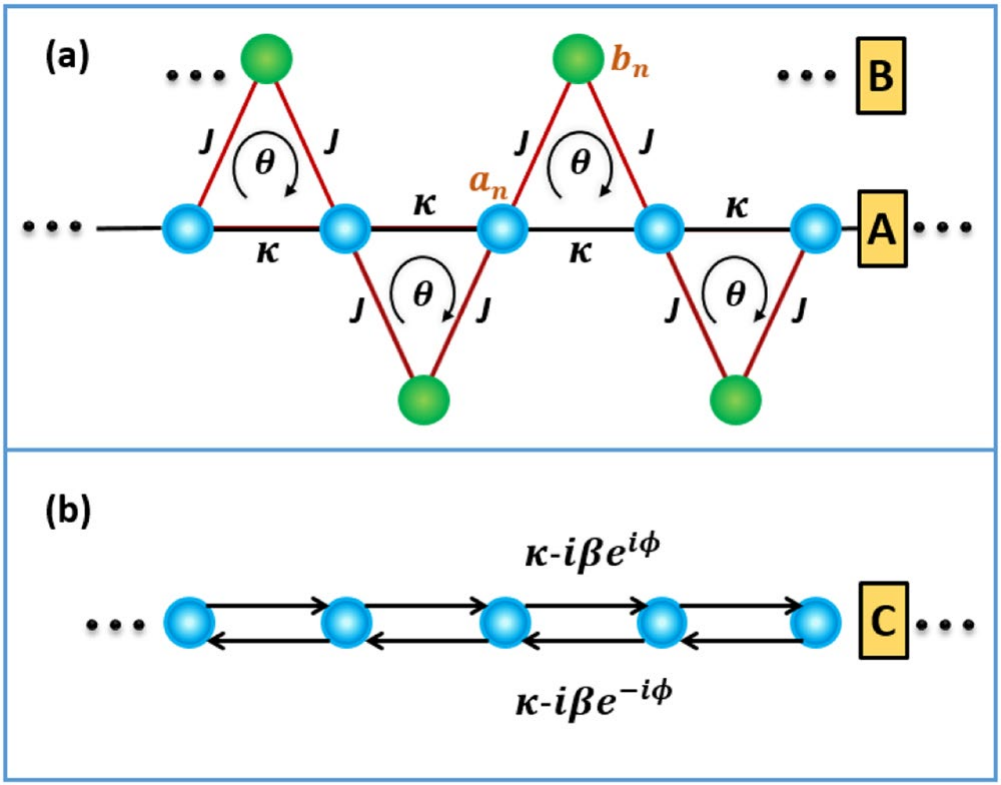
(**a**) Schematic illustration of dimerized sawtooth lattice. (**b**) Effective one-dimensional non-Hermitian lattice.

To implement the special non-Hermitian lattice, we consider a dimerized sawtooth lattice as shown in Fig. 1(a). Each unit cell comprises a main site *A* with on-site potential *U*_*a*,*n*_ and an auxiliary site *B* with on-site potential *U*_*b*,*n*_, where *n* denotes the index of the unit cells. The coupling between adjacent *A* and *B* sites (adjacent *A* sites) is denoted by *J* (*κ*). Practical implementations of such a binary lattice have been demonstrated in coupled-resonator optical waveguides^44,45^. In this paper, we consider an array of microring resonators for concreteness. Then, the real part of the potential *V*_*a* (*b*),*n*_ = Re[*U*_*a* (*b*),*n*_] denotes the resonance frequency of the *n*th main (auxiliary) resonator, while the imaginary part *γ*_*a* (*b*),*n*_ = Im[*U*_*a* (*b*),*n*_] denotes the loss or gain rate. For simplicity, we assume in the following *U*_*a*,*n*_ = *U*_*a*_ = *V*_*a*_ + *iγ*_*a*_ and *U*_*b*,*n*_ = *U*_*b*_ = *V*_*b*_ + *iγ*_*b*_. Let *a*_*n*_ and *b*_*n*_ denote the field amplitudes of sites *A* and *B* in the *n*th unit cell, respectively. Then, the evolution equations of the system under the tight-binding approximation are given by

1 $$i\frac{d{a}_{n}}{dt}={U}_{a}{a}_{n}+\kappa ({a}_{n+1}+{a}_{n-1})+J({e}^{i\theta }{b}_{n}+{e}^{-i\theta }{b}_{n-1}),$$

2 $$i\frac{d{b}_{n}}{dt}={U}_{b}{b}_{n}+J({e}^{i\theta }{a}_{n+1}+{e}^{-i\theta }{a}_{n}),$$

where *θ* is the effective Peierls phase arising from the synthetic magnetic field. In a system of charged particles, the Peierls phase is introduced by exposing the system to an actual magnetic field. However, in the case of uncharged particles such as cold atoms and photons, the Peierls phase can be obtained by artificially engineering the synthetic magnetic field. To date, synthetic magnetic fields have been successfully created for photons with various technologies^39,46–49^. In particular, the introduced Peierls phase can be dynamically controlled via electro-optical modulations^47^ and optomechanical interactions^49^.

According to the energy band theory^50,51^, if ∣*U*_*b*_ − *U*_*a*_ ± 2*κ*∣ ≫ *J*, we approximately have

3 $$0={U}_{b}{b}_{n}+J({e}^{i\theta }{a}_{n+1}+{e}^{-i\theta }{a}_{n}),$$

which implies that the auxiliary sites *B* can be eliminated adiabatically when we assume that the structure is excited in sublattice A^43,52^. In fact, similar elimination methods have been widely used in other systems such as atomic ones^53,54^. By substituting Eq. 3 back to Eq. 1 according to the adiabatic elimination, we attain the effective evolution equation for the sublattice A with ${a}_{n}^{eff}$

4 $$i\frac{d{a}_{n}^{eff}}{dt}={U}_{eff}{a}_{n}^{eff}+{J}_{1}{a}_{n+1}^{eff}+{J}_{2}{a}_{n-1}^{eff},$$

where *U*_*eff*_ = *U*_*a*_ − 2*J*^2^/*U*_*b*_ is the effective on-site potential of *A*, *J*_1_ = *κ* − *J*^2^*e*^2*iθ*^/*U*_*b*_ and *J*_2_ = *κ* − *J*^2^*e*^−2*iθ*^/*U*_*b*_ are the effective couplings in two opposite directions which clearly break the time-reversal symmetry. For convenience, we removed the real part of *U*_*eff*_ by setting *V*_*a*_ = 0 because a nonvanishing real part does not deform the energy band. Note that if *V*_*b*_ = 0, the non-Hermitian couplings *κ* − *iβe*^±2*iθ*^ with *β* ≡ − *J*^2^/*γ*_*b*_ can be obtained, the imaginary parts of which depend on the phase *θ*. Then, as shown in Fig. 1(b), the sawtooth lattice is equivalent to a non-Hermitian one *C* of amplitude *c*_*n*_ (${c}_{n}\equiv {a}_{n}^{eff}$). This case is considerably different from common Hermitian lattices with ${\kappa }_{nm}={\kappa }_{mn}^{* }$, which will be discussed in the next section. The evolution equation of the non-Hermitian lattice is given by

5 $$i\frac{d{c}_{n}}{dt}=-\,i\gamma {c}_{n}+(\kappa -i\beta {e}^{i\varphi }){c}_{n+1}+(\kappa -i\beta {e}^{-i\varphi }){c}_{n-1}$$

with *ϕ* = 2*θ* and *γ* = 2*β* − *γ*_*a*_. Note that the Peierls phase *ϕ* in Eq. 5 cannot be eliminated by any gauge transformation; hence, the light transport may depend on the phase.

By assuming the solutions to Eq. 5 in the form of *c*_*n*_ = *C*exp(*iqn* − *iEt*), the energy band of the lattice can be given by

6 $$E(q)=2\kappa \cos (q)-2i\beta \cos (q+\varphi )-i\gamma ,$$

where  − *π* ≤ *q* ≤ *π* is the Bloch wave number (quasi-momentum) in the first Brillouin zone, with  − *π* < *q* < 0 (0 < *q* < *π*) corresponding to a right-(left-)going wave. Clearly, the imaginary part of the energy describing the absorption or amplification can be modified by the phase *ϕ*, while the real part describing the dispersion relation is independent of *ϕ*. According to Eq. 6, the condition for lossless transport is given by the phase matching relation *q* + *ϕ* = ± *π*.

Using Eq. 6, one can obtain the group velocity

7 $${v}_{g}={\rm{Re}}\left(\frac{dE}{dq}\right)=-\,2\kappa \ \sin (q)$$

and the diffraction coefficient

8 $$D=-{\rm{Re}}\left(\frac{{d}^{2}E}{d{q}^{2}}\right)=2\kappa \ \cos (q)$$

of a wave in the non-Hermitian lattice. Note that *γ* = *β* = 0 corresponds to a Hermitian lattice, while a purely dissipative optical system requires *γ* ≥ 2*β* (*γ*_*a*_ ≤ 0)^43^.

**Figure 2:**
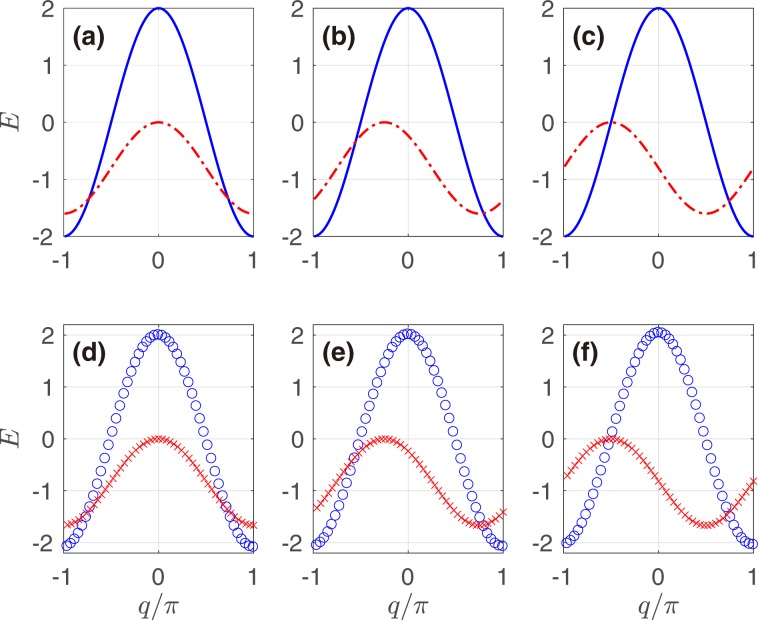
Real (blue solid line) and imaginary (red dot-dashed line) parts of q-space energy bands of the non-Hermitian lattice C with (**a**) *ϕ* = −*π*; (**b**) *ϕ* = −3*π*/4; and (**c**) *ϕ* = −*π*/2 for *γ* = 2*β* = 0.8*κ*. Real (blue circle) and imaginary (red cross) parts of the upper q-space energy bands of the sawtooth lattice A + B with (**d**) 2*θ* = −*π*; (**e**) 2*θ* = −3*π*/4; and (**f**) 2*θ* = −*π*/2 for *U*_*a*_ = 0, *U*_*b*_ = −40*iκ* and *J* = 4*κ*. The energy scale is in arbitrary units.

 Figures 2(a–c) plot the q-space energy bands of the non-Hermitian lattice C with different values of phi, while Fig. 2(d–f) show the upper q-space energy bands focused on of the sawtooth lattice A + B for comparison. The high coincidence of band structures of the two cases proves that the adiabatic elimination used is quite reasonable when the elimination condition is met involving enough large ∣*U*_*a*_-*U*_*b*_∣. As predicted in Eq. 6 and shown in Fig. 2, the imaginary part (especially the position of the maximum Im(*E*)_*max*_ = 0) may change with the phase *ϕ*, but the real part remains invariable. In ref. ^43^, the imaginary part of the energy band was fixed and had two symmetric local maximums, leading to phenomena that were obviously different from ours discussed in the next section, although the relative position between the real and imaginary parts could also be controlled.

## Results and Discussion

### Robust unidirectional light transport

The spreading dynamics of excitations in the Hermitian and non-Hermitian lattices can be studied by examining the spatial-time evolution of the normalized field amplitude ∣*ρ*_*n*_(*t*)∣^35,36,43^, i.e.,

9 $${\rho }_{n}(t)=\sqrt{\frac{| {c}_{n}(t){| }^{2}}{{\sum }_{n}| {c}_{n}(t){| }^{2}}},$$

for the single-site excitation *c*_*n*_(0) = *δ*_*n*,0_ and the Gaussian excitation

10 $${c}_{n}(0)\propto \exp \left[-\frac{{(n-{n}_{0})}^{2}}{{w}_{0}^{2}}+i{q}_{0}n\right],$$

where *n*_0_, *q*_0_, and *w*_0_ denote, respectively, the incident site, initial wave number and width of the Gaussian wave packet.

**Figure 3:**
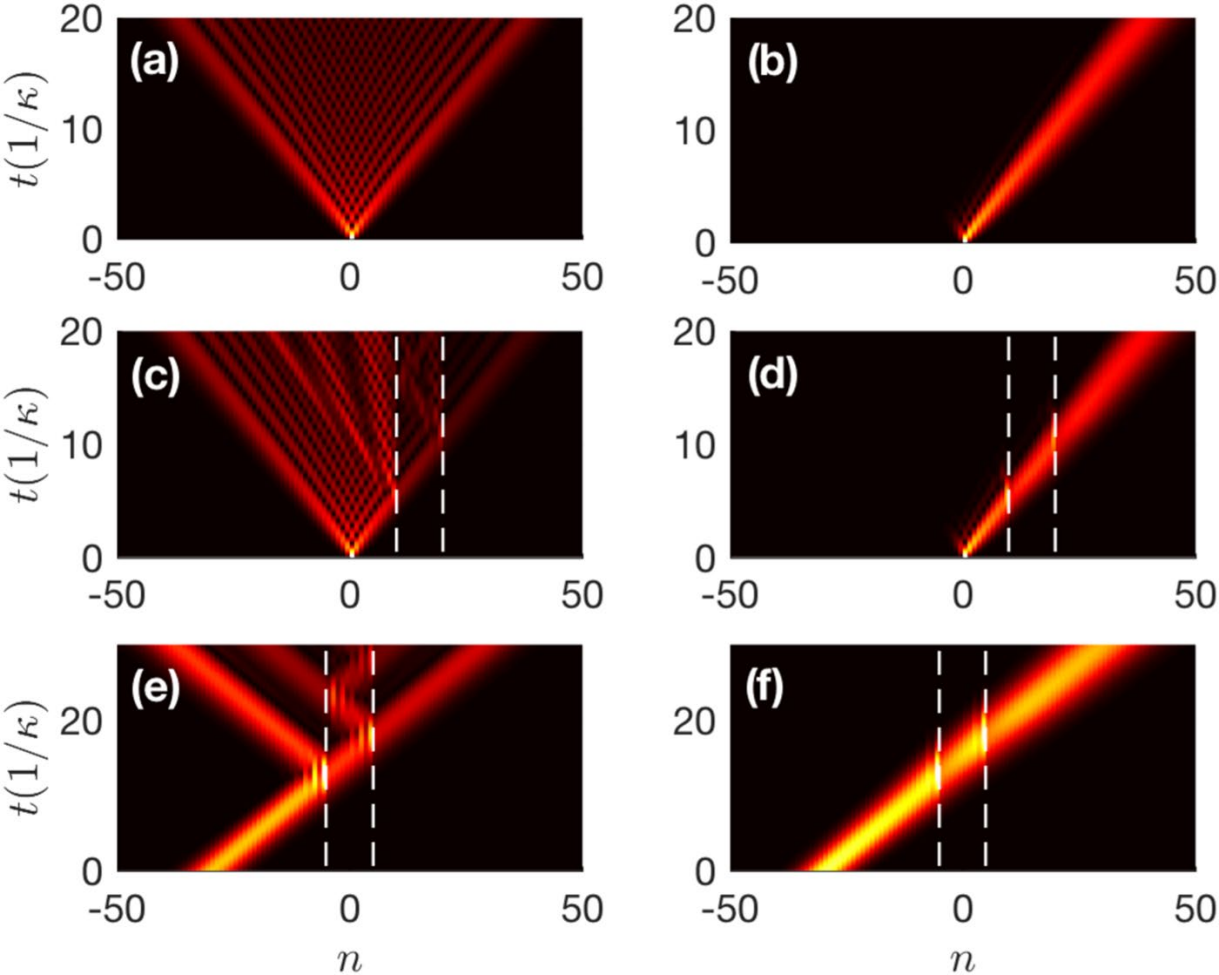
Dynamical evolutions of ∣*ρ*_*n*_(*t*)∣ for single-site (Gaussian) excitation in the Hermitian lattice (*γ* = *β* = 0) in (**a**,**c**,**e**) and the non-Hermitian lattice (*γ* = 2*β* = 0.8*κ*) with *ϕ* = −*π*/2 in (**b**) and (**d**,**f**). The dynamics in the Hermitian lattice do not depend on *ϕ*. The white dashed lines denote the defects *V*_*c*_ = 2*κ* at the sites *n* = 10, 20 (*n* = ±5) in (**c**,**d**) [in (**e**,**f**)]. The initial conditions are *c*_*n*_(0) = *δ*_*n*,0_ for the single-site excitation and *n*_0_ = −30, *w*_0_ = 5, *q*_0_ = −*π*/2 for the Gaussian excitation.

We consider first an uniform lattice. For single-site excitation, the incident wave include all Bloch wave numbers in the first Brillouin zone, exhibiting a ballistic propagation^55^ in a common Hermitian lattice, as shown in Fig. 3(a). In Fig. 3(b), however, the incident wave exhibits unidirectional propagation with the velocity mainly corresponding to Im(*E*) = 0 through the non-Hermitian lattice *C*. The propagation direction, group velocity, and diffusion of waves vary periodically with *ϕ* according to Eqs. 7 and 8. The physical reason is that, only the wave component with *q* = ± *π* − *ϕ* can propagate without loss as mentioned above, the other components decay rapidly during propagation, corresponding to *evanescent* waves. In particular, the group velocity reaches the maximum *v*_*g*_ = 2*κ* and the diffusion becomes weakest if *ϕ* = ± *π*/2, with the sign of *ϕ* determining the propagation direction. However, one can select the lossless wave component by adjusting the phase *ϕ* according to the phase matching relation. Based on this, the non-Hermitian lattice can serve as a frequency-selectable filter, with which outgoing waves of desired wave numbers can be obtained.

It is worth mentioning that the unidirectional light transport is robust against lattice defects. To prove this, in Fig. 3(c–f), we introduce defects *V*_*def*_ = *V*_*c*_(*δ*_*n*,10_ + *δ*_*n*,20_) [*V*_*def*_ = *V*_*c*_(*δ*_*n*,−5_ + *δ*_*n*,5_)] with the purely real amplitude *V*_*c*_ and replace  − *iγc*_*n*_ by (*V*_*def*_ − *iγ*)*c*_*n*_ in Eq. 5. Then dynamic evolution can be observed in the non-Hermitian (Hermitian) lattice with defects by setting *γ* = 2*β* = 0.8*κ* (*γ* = *β* = 0). It can be found that the wave is immune to defects in the non-Hermitian lattice, i.e., the transport is highly robust, while the wave undergoes multiple scatterings between the defects in the Hermitian one, as in a Fabry-Perót cavity. Moreover, the robust unidirectional transport can also be observed for the Gaussian excitation essentially including only a narrow range of Bloch wave numbers. Compared with the Hermitian case shown in Fig. 3(e,f) shows that the Gaussian wave with initial wave number *q*_0_ is immune to defects in the non-Hermitian lattice with the robustness becoming strongest when *ϕ* = − (*q*_0_ ± *π*).

The underlying physics is as follows. For the common Hermitian lattice, the purely real energy band is symmetric with respect to *q* = 0, i.e., the energy band is degenerate. In this case, the reflected wave with wave number ${q}^{^{\prime} }=-{q}_{0}$ and energy $E({q}^{^{\prime} })=E({q}_{0})$ is allowed to propagate due to the elastic scattering, with *q*_0_ and *E*(*q*_0_) being the wave number and energy of the incident wave, respectively. In the non-Hermitian lattice with *ϕ* = ±*π* [see Fig. 2(a)], the energy band remains degenerate so that the reflected wave can still be observed. When *ϕ* ≠ ± *π*, however, the degeneracy of the complex energy band may be broken by the motion of the imaginary part [see Fig. 2(b,c)], and thus $E({q}^{^{\prime} })=E({q}_{0})$ has no real solution except for ${q}^{^{\prime} }={q}_{0}$, implying that the reflected wave becomes evanescent^36,43^. Therefore, the non-Hermitian lattice can support unidirectional light transport without reflection and immune to defects.

**Figure 4:**
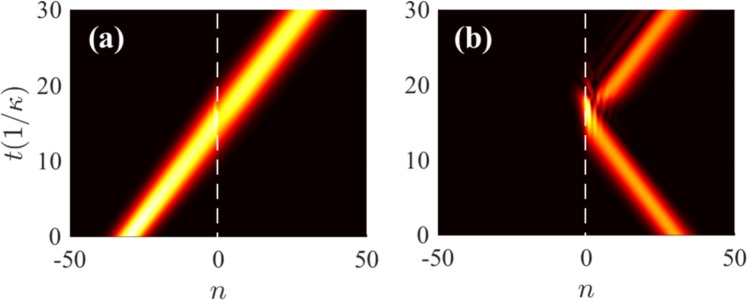
Dynamical evolutions of ∣*ρ*_*n*_(*t*)∣ for Gaussian excitation in the heterostructure with (**a**) *n*_0_ = −30, *q*_0_ = −*π*/2 (left-incident wave) and (**b**) *n*_0_ = 30, *q*_0_ = *π*/2 (right-incident wave). The white dashed lines denote the interface site *n* = 0. The other parameters are the same as those in Fig. 3.

On the basis of the non-Hermitian lattice, we construct a heterostructure that is formed by connecting a non-Hermitian lattice *C* at the left with a Hermitian lattice at the right. As shown in Fig. 4(a), the left-incident wave with initial wave number *q*_0_ shows strong transmission from left to right and no reflection when *ϕ* = − (*q*_0_ ± *π*). However, it is found in Fig. 4(b) that, any right-incident wave cannot penetrate the left non-Hermitian part and is almost totally reflected back with the same phase. This tunable unidirectional transmissionless phenomenon is the key to realizing optical diodes.

### Light trapping and reversal

**Figure 5:**
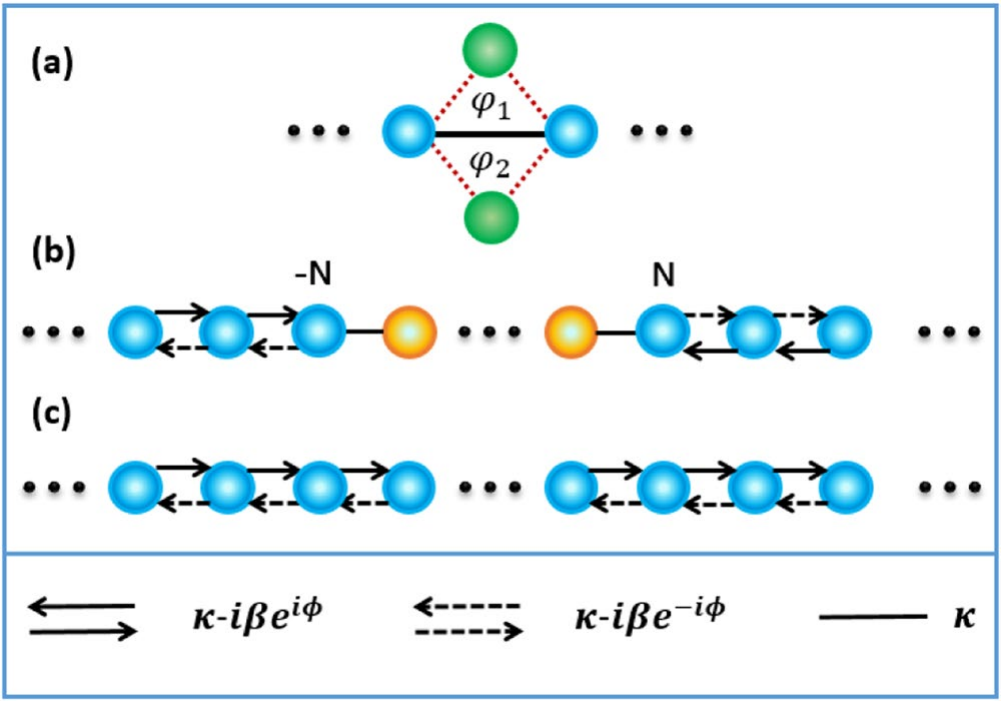
Schematic illustration of (**a**) the implementation scheme of the switchable middle part, (**b**) the sandwich structure used for light trapping, and (**c**) the uniform non-Hermitian lattice used for light retrieving. The green circles denote the auxiliary sites, while the blue (yellow) circles denote the main sites of the non-Hermitian (Hermitian) part. Sites *n* = ± *N* are the interfaces of this sandwich structure.

In this section, we consider a one-dimensional sandwich structure, where the two side parts are non-Hermitian lattices and the middle part can be switched between Hermitian and non-Hermitian ones by adjusting the phases. Specifically, in the middle part as shown in Fig. 5(a), we introduce two identical auxiliary sites (an upper one and a lower one) between every two adjacent main sites. The coupling rates between each main site and its adjacent auxiliary sites are the same, while the effective Peierls phases arising from the upper and lower auxiliary sites are *φ*_1_ and *φ*_2_, respectively. When *φ*_1_ = −*φ*_2_ = ±*π*/2, the middle part is Hermitian, i.e., the effective coupling between adjacent main sites is real due to the offset between the upper and lower auxiliary sites; when *φ*_1_ = *φ*_2_ = *ϕ*, however, the middle part becomes non-Hermitian as shown in Fig. 1(b).

Firstly, the sandwich structure is prepared as shown in Fig. 5(b), i.e., the tunable middle part is Hermitian and the effective Peierls phases of the two side parts are opposite. The sandwich structure is excited by a right-going Gaussian wave with initial wave number *q*_0_ < 0, which is input upon the left non-Hermitian part with matching phase *ϕ*_0_ = − (*q*_0_ + *π*). Taking all above conditions into account, the evolution equations of the sandwich structure can be written as

11 $$i\frac{d{c}_{n}}{dt}=\left\{\begin{array}{l}-i\gamma {c}_{n}+{\nu }_{1}{c}_{n+1}+{\nu }_{2}{c}_{n-1}\,\,\,\,\,\,n < -\,N,\\ i\xi {c}_{n}+\kappa {c}_{n+1}+{\nu }_{2}{c}_{n-1}\,\,\,\,\,\,\,\,\,\,n=-\,N,\\ \kappa ({c}_{n+1}+{c}_{n-1})\,\,\,\,\,\,\,\,\,\,\,\,\,\,\,\,\,\,\,\,\,\,\,| n|  < N,\\ i\xi {c}_{n}+{\nu }_{2}{c}_{n+1}+\kappa {c}_{n-1}\,\,\,\,\,\,\,\,\,\,\,\,\,n=N,\\ -i\gamma {c}_{n}+{\nu }_{2}{c}_{n+1}+{\nu }_{1}{c}_{n-1}\,\,\,\,\,\,\,\,\,n > N,\\ \end{array}\right.$$

with ${\nu }_{1,2}=\kappa -i\beta {e}^{\pm i{\phi }_{0}}$. Sites *n* = ± *N* are the two interfaces between the middle and side parts. According to Eq. 11, Im[*E*_*N*_(*q*_0_)] = Im[*E*_−*N*_(− *q*_0_)] = Im[*iβ* exp(2*iq*_0_)]. This implies that within the middle part, whenever the wave is scattered at the interface sites *n* = ± *N* owing to the abrupt changes in the energy band, it may suffer attenuation or amplification which is determined by *q*_0_. The cumulative effect of the attenuation or amplification is considerable because the wave may be scattered a few times before complete decay. To offset this effect, we introduce additional imaginary potentials *iξ* at the interface sites. Here, we consider that the wave of *q*_0_ = − *π*/2 is input from the left side. Thus, owing to the attenuation effect Im[*iβ* exp(2*iq*_0_)] = −*β*, we assume *ξ* = *β* which is equivalent to a finite gain compensation.

**Figure 6:**
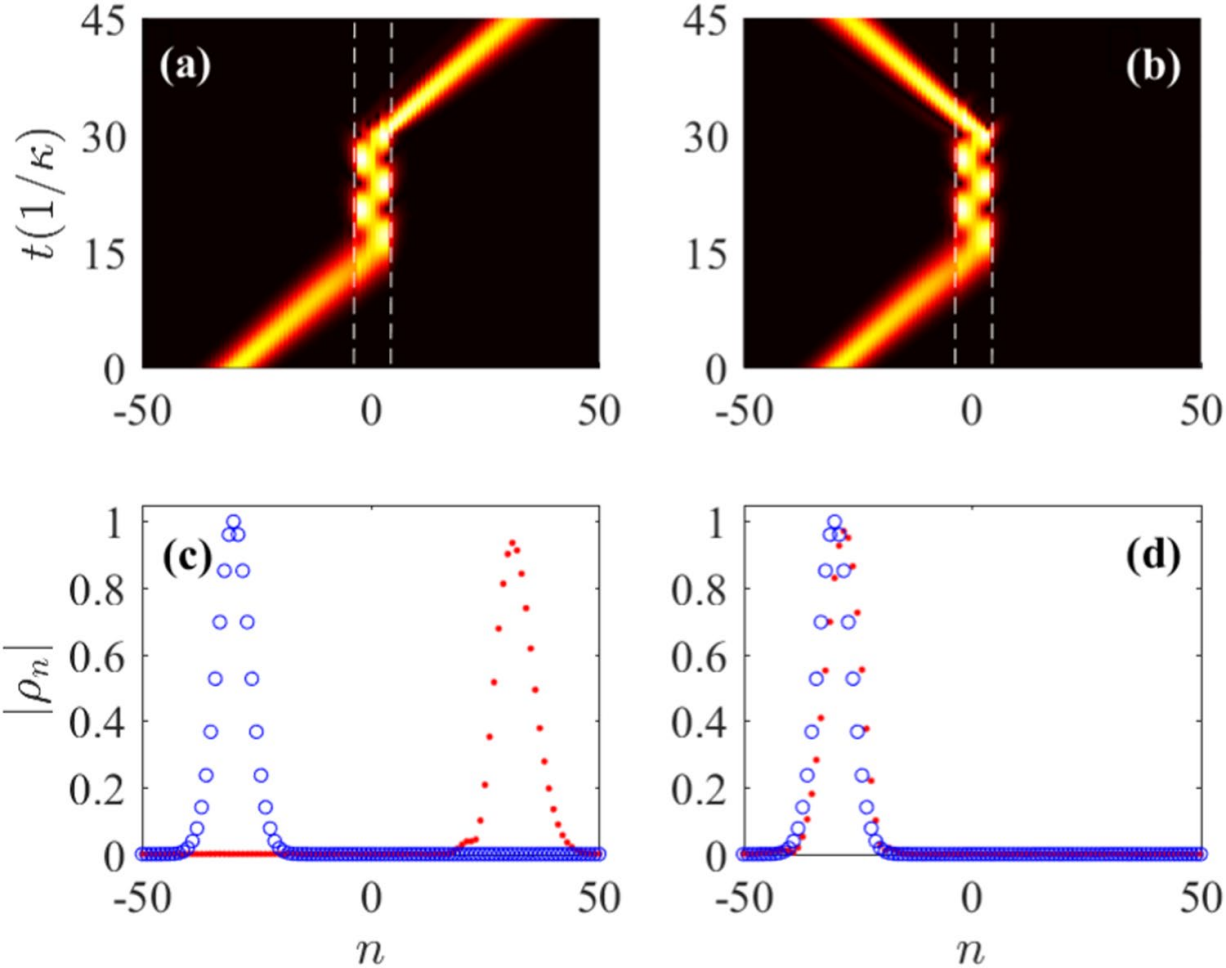
Dynamical evolutions of ∣*ρ*_*n*_(*t*)∣ for Gaussian excitation in the sandwich structure with (**a**) *ϕ*_*r*_ = − *π*/2 and (**b**) *ϕ*_*r*_ = *π*/2. The normalized amplitude profiles ∣*ρ*_*n*_∣ of the incident (blue circles) and retrieval (red dots) waves in (**a**,**c**) and in (**b**,**d**). The white dashed lines in (**a**,**b**) denote the interface sites with *N* = 3. Here, we assume *t*_*r*_ = 30/*κ* and *ξ* = *β*. The other parameters are the same as those in Fig. 3.

As shown in Fig. 6(a), the left-incident wave propagates unidirectionally through the left non-Hermitian part. Once entering the middle Hermitian part, the wave is scattered back and forth between the two interface sites so that the light is captured. Then, by adjusting all Peierls phases to *ϕ*_*r*_ = *ϕ*_0_ at *t* = *t*_*r*_ (*t*_*r*_*κ* = 30, the sandwich structure is switched to a uniform non-Hermitian lattice being identical with the initial left part, as shown in Fig. 5(c). The evolution equation thus becomes

12 $$i\frac{d{c}_{n}}{dt}=(\kappa +i\beta {e}^{i{\varphi }_{r}}){c}_{n+1}+(\kappa +i\beta {e}^{-i{\varphi }_{r}}){c}_{n-1}+i[\xi \left({\delta }_{n,N}+{\delta }_{n,-N}\right)-\gamma ]{c}_{n}$$

with *ξ* = *β*. In this way, the light can be retrieved at the right side. Moreover, as shown in Fig. 6(b), by changing all phases to *ϕ*_*r*_ = −*ϕ*_0_ in the retrieving step, it shows light reversal at the left (incident) side owing to phase matching^56^. Fig. 6(c,d) depict the normalized amplitude profiles of the incident and retrieval waves in Fig. 6(a,b), respectively. We can find that the retrieval waves maintain the Gaussian shape in both cases, i.e., the trapping scheme is nearly shape-preserving.

Physically, the wave oscillation in the middle part occurs because reflected waves are allowed to propagate in the Hermitian lattice. However, by setting opposite Peierls phases for the two side parts, ${E}^{^{\prime} }({q}^{^{\prime} })=E(q)$ has no real solution except for ${q}^{^{\prime} }=q$, where $E\ ({E}^{^{\prime} })$ and $q\ ({q}^{^{\prime} })$ are the energy and Bloch wave number of the Hermitian (non-Hermitian) part, respectively, so that wave transmission from the Hermitian part to the non-Hermitian part is prevented due to the evanescent transmitted waves. Once the sandwich structure is switched to a uniform non-Hermitian lattice, the retrieval light can propagate robustly and unidirectionally with the direction determined by the retrieving phase *ϕ*_*r*_ = ±*π* −*q*_0_. Moreover, the tunable light trapping scheme allows the flexible control of the trapping duration and region (the length of the middle part). This scheme obviously increases the interaction time between light and matter with low loss and thus can provide a excellent platform for the photonic quantum modulation^57^.

**Figure 7:**
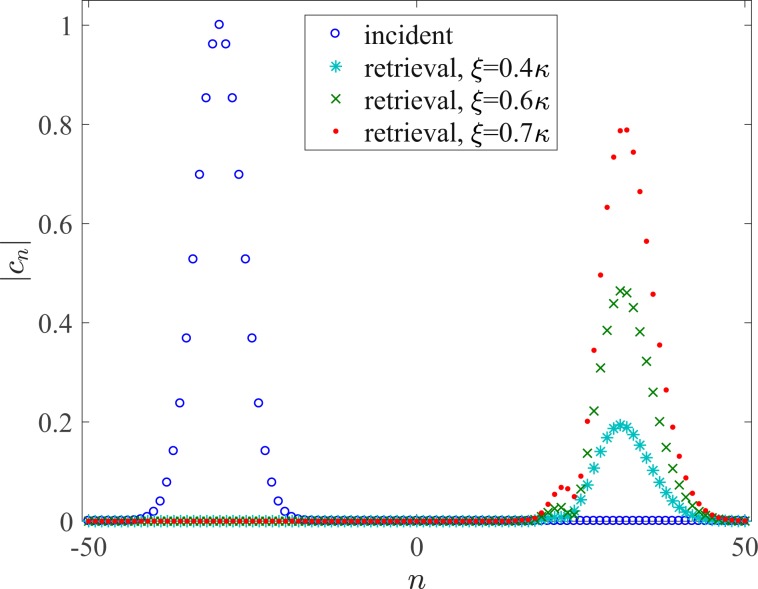
Actual amplitude profiles ∣*c*_*n*_∣ of the incident and retrieval waves with different offset coefficients *ξ*. All parameters except for *ξ* are the same as those in Fig. 3.

Finally, to examine the effect of the additional imaginary potentials *iξ* on the retrieval efficiency, we plot in Fig. 7 the actual amplitude profiles of the incident and retrieval waves of the light trapping process in Fig. 6(a) with different offset coefficients *ξ*. Clearly, although we have introduced the gain compensation with *ξ* = *β* = 0.4*κ* at the interface sites as discussed above, the actual amplitude of the retrieval wave is still much smaller than that of the incident one. This is because, for a Gaussian wave packet, some components with the wave number not satisfying *ϕ* + *q*_0_ = ±*π* may undergo different levels of loss in the non-Hermitian parts. However, by increasing *ξ* properly, the actual amplitude of the retrieval wave can be significantly enhanced with a satisfactory shape-preserving effect. Moreover, the efficiency can also be increased by introducing proper gain throughout the entire sample. Thus, the light trapping scheme can be optimized by means of finite gain compensations.

## Conclusions

In summary, we proposed a one-dimensional lattice with special non-Hermitian coupling, the imaginary part of which can be modulated by the effective Peierls phase arising from a synthetic magnetic field. Such a non-Hermitian lattice can be achieved by reducing a dimerized sawtooth lattice containing an array of auxiliary sites via proper adiabatic elimination. We found that this non-Hermitian lattice can support robust unidirectional light transport that is reflectionless and immune to defects. Moreover, this lattice can serve as a tunable filter for selecting waves with desired wave numbers. To explore more novel applications, we further built two structures involving the non-Hermitian lattice, namely a heterostructure and a sandwich structure. The heterostructure could be used to realize unidirectional transmissionless transport, i.e., an optical diode scheme, while light trapping and reversal could be realized and controlled through phase modulations of the sandwich structure. This scheme can obviously increase the interaction time between light and matter. By introducing finite gain, the efficiencies of the trapping and reversal processes could be increased significantly. We hope that the results can not only open a new path for unconventional wave transport but also provide a promising platform for photon quantum modulation.
